# Weiqi Decoction Attenuated Chronic Atrophic Gastritis with Precancerous Lesion through Regulating Microcirculation Disturbance and HIF-1*α* Signaling Pathway

**DOI:** 10.1155/2019/2651037

**Published:** 2019-06-20

**Authors:** Jing Yin, Jinyu Yi, Chun Yang, Bo Xu, Jiang Lin, Hongyi Hu, Xiaojun Wu, Hailian Shi, Xiaoyan Fei

**Affiliations:** ^1^Longhua Hospital, Shanghai University of Traditional Chinese Medicine, Shanghai 200032, China; ^2^Shanghai Key Laboratory of Compound Chinese Medicines, Institute of Chinese Materia Medica, Shanghai University of Traditional Chinese Medicine, Shanghai 201203, China

## Abstract

**Aim:**

Chronic atrophic gastritis (CAG), the precancerous lesions of gastric cancer, plays an important role in the stepwise process of gastric cancer. The ancient Chinese medicine believes in that Qi deficiency and blood stasis are involved in the pathogenesis of CAG. Weiqi decoction, a classical formula from Longhua Hospital, could supplement Qi and activate blood circulation of human beings and has been used for treating CAG in clinic over twenty years. The study aims to clarify the effect and underlying molecular mechanism of Weiqi decoction on CAG rats.

**Methods:**

Forty-eight male Wistar rats were divided randomly into six groups: control group, model group, folic acid group, and WQD-treated groups at doses of 4 g/kg, 2 g/kg, and 1 g/kg, with eight rats in each group. MNNG and saturated NaCl were used to induce CAG rat with precancerous lesion (intestinal metaplasia and dysplasia). After 40 weeks, gastric mucosal blood flow was measured using Laser Doppler Flowmetry. The pathological changes of the gastric mucosa were identified by H&E staining and AB-PAS staining. The protein expression of COX-2, HIF-1*α*, VEGFR1, VEGFR2, Ki67, and cleaved caspase 3 in the gastric tissues was measured by western blotting approach. Gene expression of COX-2, HIF-1*α*, VEGF, VEGFR1, VEGFR2, Ang-1, and Ang-2 was detected by using Quantitative PCR method. The PGE2 concentrations in serum were detected by ELISA method. The protein expression of Ki67 in gastric mucosa was also detected by immunohistochemistry.

**Results:**

Compared with control rats, atrophy and intestinal metaplasia as well as the microcirculation disturbance of gastric mucosa were induced in the stomach of CAG rats identified by the H&E and AB-PAS staining as well as microcirculation measurement, which could be significantly attenuated by WQD treatment. Moreover, compared with the control group, the protein and gene expression of COX-2, HIF-1*α*, VEGFR1, and VEGFR2 in gastric tissues of pylorus was obviously increased and the serum PGE2 level was significantly deceased in CAG rats, which could be significantly counteracted by WQD administration. However, the gene expression of Ang-1 and Ang-2 was not significant difference between control rats and CAG rats, and WQD also had no significant effect on the gene expression of Ang-1 and Ang-2. Furthermore, the increased cell proliferation marked by upregulated protein expression of Ki67 and decreased cell apoptosis marked by downregulated protein expression of cleaved caspase 3 in stomach of pylorus in CAG rats were obviously reversed by WQD treatment.

**Conclusion:**

WQD attenuated CAG with precancerous lesion through regulating gastric mucosal blood flow disturbance and HIF-1*α* signaling pathway.

## 1. Introduction

Gastric cancer (GC) is the fifth most common cancer and the third leading cause of cancer death over the world. More than 70% of gastric cancer cases occur in the development countries. And half of all the cases in the world occur in eastern Asia mainly in China [1, 2]. Chronic atrophic gastritis (CAG) with precancerous lesion (intestinal metaplasia and dysplasia) plays a very important role in the pathological progress of GC; however, there is still no standard therapy for it. Thus, it is very urgent to develop new drugs for treating CAG with precancerous lesion.

In modern medicine,* Helicobacter pylori* (Hp) infection and autoimmunity are the main inducement of CAG [3]. The conventional therapy is eradicating Hp infection in CAG patients. At present, the triple therapy of proton pump inhibitor (PPI) and two antibiotics is the first-line treatment recommended [4–6]. However, resistance to antibiotics of Hp has increased year by year [7, 8]. Furthermore, the effect of the classical treatments on the atrophy and intestinal metaplasia is still controversial. In the theory of traditional Chinese medicine, CAG belongs to stomach pain, bloating, abdominal distention, etc., resulting from Qi deficiency and blood stasis [9, 10]. Compared to western medicine, traditional Chinese medicine prescribed as a formula for patients with FD and CAG get a pleasant therapeutic effect [11–14].

Weiqi decoction (WQD), an empirical formula sourced Longhua Hospital affiliated to Shanghai University of Traditional Chinese Medicine, has been used for functional dyspepsia (FD) and CAG treatment by raising Qi, smoothing the blood, and harmonizing the stomach for over twenty years in clinic [15–19]. It consists of* Radix Angelicae Sinensis *(12 g),* Radix Astragali *(12 g),* Radix Codonopsis *(15 g),* Rhizoma Curcumae *(15 g),* Fructus Aurantii *(15 g),* Fructus Akebiae* (15 g), and* Herba Taraxaci *(30 g). Previous researches have already confirmed the therapeutic effect of WQD on CAG with precancerous lesion (intestinal metaplasia and dysplasia) in patients [15–17]. In vitro, WQD could also promote cell apoptosis of gastric cancer cells in a mitochondria-dependent manner [16, 20] and inhibit cell growth and tube formation of HUVECs, indicating inhibiting angiogenesis in the progress of gastric cancer [21]. Although few previous researches proved microcirculatory disorder truly existed in CAG [13], the effect of WQD on gastric blood circulation of CAG and the underlying molecular mechanism of WQD on CAG with precancerous lesion has not been well-clarified yet.

Angiogenesis and microcirculation disorders were found in both animal and CAG patients [22–24]. The hypoxia-inducible factor-1*α* (HIF-1*α*) signaling pathway plays a vital role in blood microcirculation disorders including ischemic, hypoxia, inflammation, and tumor angiogenesis [25–28]. As a transcription factor, HIF-1*α* directly regulates gene expressions of many target proteins involved in cancer angiogenesis, especially under hypoxia, and vascular endothelial growth factor (VEGF) is the target gene which is closely associated with angiogenesis [29]. Gastric mucosa injury could induce upregulation of HIF-1*α*, VEGF, and COX-2 [30]. And cyclooxygenase-2 (COX-2) could induce overexpression of HIF-1*α* and VEGF [25]. In present study, the effects of WQD on gastric blood microcirculation and HIF-1*α* signaling pathway in stomach tissue as well as PGE2 level in serum and expression of cell proliferation- and apoptosis-related proteins such as Ki 67 and cleaved caspase 3 in stomach tissue of CAG rats were investigated.

## 2. Methods

### 2.1. Materials

N-Methyl-N'-nitro-N-nitrosoguanidine (95%) and sodium chloride (99.8%) were purchased from Sinopharm Chemical Reagent Co. Ltd (Shanghai, China). Folic acid (16101812) was obtained from Changzhou Pharmaceutical factory Co., Ltd. (Jiangsu, China). The Weiqi decoction (cat #: 1708001) was purchased from Longhua Hospital affiliated to Shanghai University of Traditional Chinese Medicine. The drug ratio, voucher specimen number, method of preparation of WQD, and HPLC analysis of WQD could be found in our previous research [20]. All the voucher specimen of herb plants in WQD was verified and deposited in Shanghai Research and Development Center for Standardization of Chinese Medicine. The antibodies against VEGFR1 (cat: ab184784, 1:2500), Ki67 (cat: ab16667, 1:200), and PGE2 ELISA kit (cat: ab136948) were purchased from Abcam plc. (Shanghai, China). The antibodies against COX-2 (cat: 12282, 1:1000) and GAPDH (cat: 5174, 1:5000) were provided by Cell Signaling Technology, Inc. (Shanghai, China). HIF-1*α* (cat: 610958, 1:500) antibody was supplied from Becton, Dickinson and Company (Shanghai, China).

### 2.2. Animals

Forty-eight male Wistar rats with 160-180 g were kept in the Laboratory Animal Center of Shanghai University of Traditional Chinese Medicine (TCM) (Shanghai, China). All animals were adapted for at least one week before experiments and maintained in the standard laboratory condition of temperature (25 ± 2°C), relative humidity (60 ± 2%), an alternating lighting (12 h light: 12 h dark cycle), and free access to sufficient food and water. All animal experiments were performed according to the protocols approved by Animal Care and Use Committee of Shanghai University of Traditional Chinese Medicine, which complies with international rules and policies for laboratory animal use and care as found in the European Community guidelines (EEC Directive of 1986; 86/609/EEC). All animal experiments were approved by the Institutional Ethics Committee of Shanghai University of TCM (SZY201703012).

### 2.3. Preparation of WQD

Preparation of WQD was performed as the procedure of our previous study [20]. Briefly speaking, WQD consisting of* Radix Angelicae Sinensis *(12 g),* Radix Astragali *(12 g),* Radix Codonopsis *(15 g),* Rhizoma Curcumae *(15 g),* Fructus Aurantii *(15 g),* Fructus Akebiae *(15 g), and* Herba Taraxaci *(30 g), was powered and soaked in 10 volumes 95% ethanol for 12 h and then boiled for 2 h. After that, the mixture was filtered and the liquid was collected. Afterwards, 8 volume 95% ethanol was added to the filtered powder and boiled for 2 h, then the mixture was filtered, and liquid was also collected. The same procedure was repeated for one more time. Finally, all the liquid was pooled together and evaporated under vacuum till the ethanol was recovered completely. And the liquid was dried in vacuum drying oven. Our previous HPLC analysis results showed that the contents of four active compounds in WQD were as follows: naringin, 35.92 *μ*g/mg; nobiletin, 21.98 *μ*g/mg; neohesperidin, 17.98 *μ*g/mg; tangeretin, 0.756 *μ*g/mg [20].

### 2.4. Animal Experiment Design and CAG with Precancerous Lesion Model Induction

Forty-eight Wistar rats were randomly divided into 6 groups (n=8 per group), namely, control group, model group, positive group (folic acid, 1.5 mg/kg), WQD-treated groups at doses of 4 g/kg, 2 g/kg, and 1g/kg, respectively. CAG rats were induced with MNNG (200 mg/kg body weight) by oral gavage at day 0 and 14 and saturated NaCl (1 ml per rat) was given three times a week by oral gavage during the first 3 weeks. Then MNNG (600 *μ*g/kg) and NaCl (1 ml per rat) were given alternately up to 40 weeks. At the same time, the drug-treated groups and positive group (folic acid) were treated with WQD and folic acid 6 times per week by intragastric gavage, respectively.

### 2.5. Measurement of the Gastric Mucosal Blood Flow (GMBF)

After overnight fasting, the rats were under anesthesia by intraperitoneal injection with 3% pentobarbital sodium (30 mg/kg body weight). After opening the abdominal wall, a fiber-optic probe of Moorlab Laser Doppler flowmeter was put on the stomach wall to measuring the blood circulation of stomach. The GMBF of three parts including the fundus, the gastric body, and the pylorus were determined, respectively. And the voltage value was recorded to represent the relative GMBF.

### 2.6. Specimen Collection

After measuring the GMBF, the rats were sacrificed and the stomach was taken out and cut along the greater curvature, washed with 0.9% sodium chloride. Then the tissue was separated into two parts. One part was stored under -80°C for western blotting and Quantitative PCR analysis; the other part was fixed with 10% formalin for pathology assay.

### 2.7. Pathology Assay

Tissue specimens fixed in 10% formalin were embedded in paraffin and then were cut into 5 *μ*m sections. All the sections of the rat specimens were conducted with hematoxylin and eosin (H&E) staining or Alcian blue and periodic acid-Schiff (AB-PAS) staining. And the images were captured with microscope (Olympus, BX61VS) and scored according to histopathological grading standard.

### 2.8. PGE2 Level Measurement

The rat blood was collected and the serum was separated by centrifuge at 4 000 rpm for 15 min at 4°C. The PGE2 level in serum was detected by ELISA method according to the manufacturer's instructions.

### 2.9. Western Blot Analysis

The stomach tissue were homogenized and lysed in CellLytic™ MT mammalian tissue lysis reagent mixed with protease inhibitor cocktails and phosphatase inhibitor cocktails on ice. The homogenate was centrifuged at 12 000 rpm for 15 min at 4°C and the supernatant was collected and stored under -80°C. Protein concentration of the supernatant was quantified by BCA assay. Then, equal amount of protein from each sample was separated by SDS-PAGE (8% or 15%) and transferred onto PVDF membrane via wet electrophoretic transfer system (Bio-Rad). Afterwards, the PVDF membranes were blocked with 5% BSA for 1 h and then washed with 1×PBST buffer, incubated with different primary antibodies at 4°C overnight. After thoroughly washed with 1×PBST, PVDF membranes were further incubated with respective secondary antibodies. Thereafter, the protein bands were visualized with ECL-prime kit and analyzed with Tanon Gis software.

### 2.10. Quantitative PCR (qPCR) Analysis

Total RNAs from the tissues were extracted by using Trizol reagent (Life Technologies). After removal of the trace amounts of DNA contamination with DNase I, the reverse transcription of total RNA into cDNA was done with the RevertAid First Strand cDNA Synthesis kit from Thermo Fisher Scientific. Quantitative PCR was performed with SYBR kit (Sigma). qPCR was performed with SYBR Premix EX Taq under the following conditions: 95°C, 30 s; then followed by 40 cycles (95°C, 5 s; 60°C, 34 s); finally 95°C, 15 s; 60°C, 1 min; 95°C, 15 s. Quantity of target genes calculated by the comparative C_t_ method was normalized to that of glyceraldehyde-3-phosphate dehydrogenase (GAPDH) (internal reference) in the same sample. The sequences for forward and reverse primers used were listed in the Table 1.

### 2.11. Immunohistochemistry (IHC) Staining

The paraffin-embedded sections were dewaxed and rehydrated with xylene and graded ethanol. The activity of endogenous peroxidase was blocked by methanol containing 3% H_2_O_2_ for 10 min and washed with 1×PBS. Then the sections were blocked with 5% BSA in PBS for 1 h at 37°C, and then sections were incubated with the primary antibody against Ki67 (1:200, Abcam) overnight at 4°C. After that, all sections were washed with 1×PBS and incubate with secondary antibody for 30 min. Visualization was performed by using diaminobenzidine (DAB) and all immune-stained sections were counter-stained with hematoxylin. The quantification of Ki 67 expression was observed under microscope (Olympus BX61VS) and analyzed with the Image Pro Plus 6.0 software.

### 2.12. Statistical Analysis

All data were expressed as mean ± SD. Difference among groups was analyzed by one-way ANOVA using Graphpad Prism 5 software with Dunnett's multiple comparison test. Difference was considered statistical significance when* p *< 0.05.

## 3. Results

### 3.1. WQD Improved Gastric Mucosal Microcirculation in CAG Rats with Precancerous Lesion

**Figure 1:**
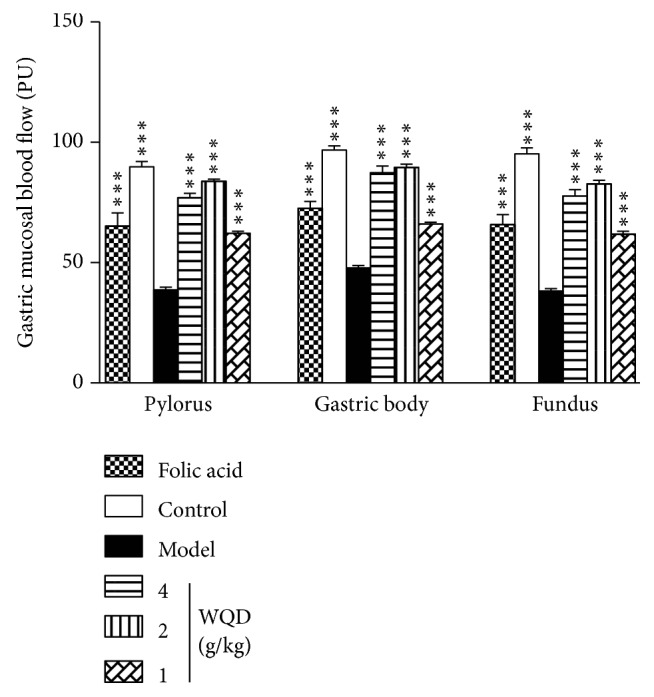
Effects of WQD on gastric mucosal microcirculation in stomach of CAG rats. Values were expressed as mean ± SD (n=6-8/group). Data were analyzed by one-way ANOVA assay. *∗∗∗*p < 0.001 vs model group.

As shown in Figure 1, after 40 weeks of CAG induction, the gastric mucosa blood flow of the fundus, gastric body and pylorus in model rats was obviously decreased compared with the normal rats (p<0.001). WQD treatment (4 g/kg, 2 g/kg, 1 g/kg) and folic acid treatment could significantly increase the gastric mucosal blood flow (p<0.001).

### 3.2. WQD Attenuated Pathological Changes in Gastric Mucosa of CAG Rats with Precancerous Lesion

**Figure 2:**
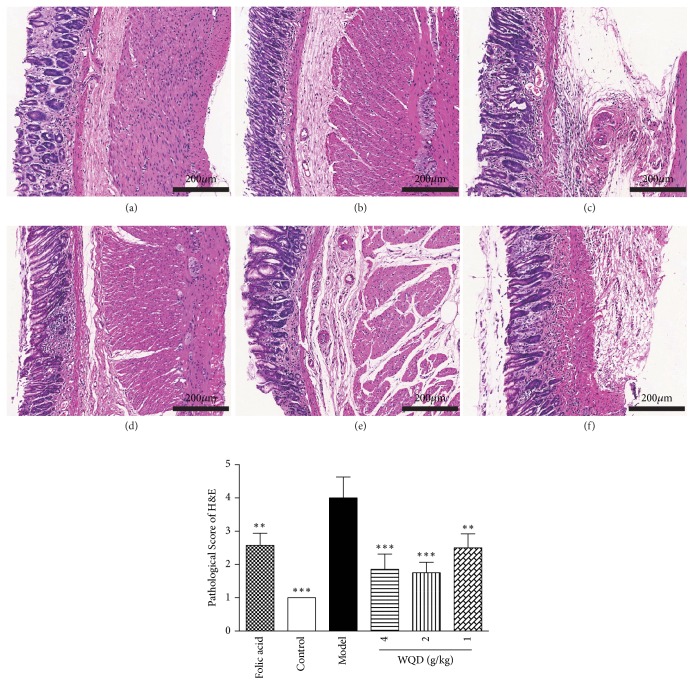
The representative pathology of gastric tissues in each group (H&E staining, 100× magnifications). (a) Folic acid group, (b) control group, (c) model group, (d) WQD 4 g/kg group, (e) WQD 2 g/kg group, and (f) WQD 1 g/kg group. Values were expressed as mean ± SD (n=6-8/group). Data were analyzed by one-way ANOVA assay. *∗∗*p < 0.01; *∗∗∗*p < 0.001 vs model group.

**Figure 3:**
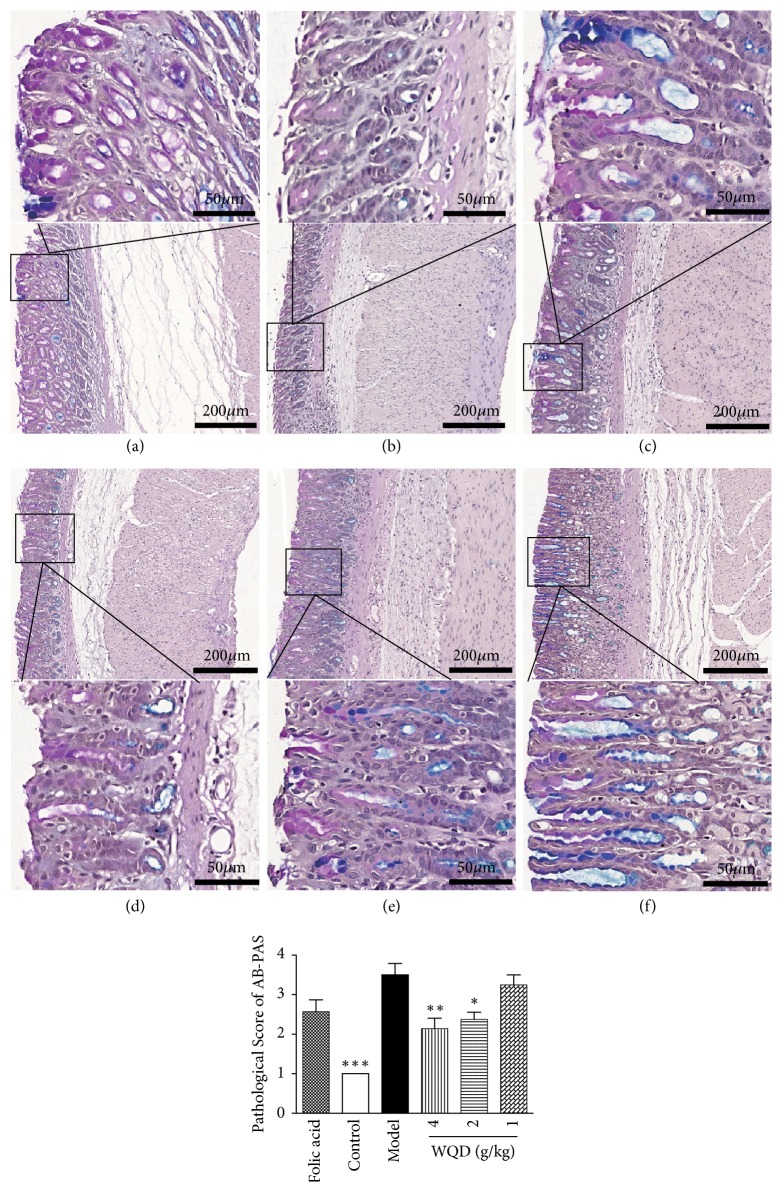
The representative pathology of gastric tissues in each group (AB-PAS staining, 100× magnifications). Gastric intestinal metaplasia changes were stained with purple and blue color. (a) Folic acid group, (b) control group, (c) model group, (d) WQD 4 g/kg group, (e) WQD 2 g/kg group, and (f) WQD 1g/kg group. Values were expressed as mean ± SD (n=6-8/group). Data were analyzed by one-way ANOVA assay. *∗* p < 0.05; *∗∗*p < 0.01 vs model group.

As shown in Figure 2, gastric glands of the normal rats were arranged in order, while in the model rats, the glands of pylorus were arranged in a disorderly manner and the size of gastric glands was different from each other. Moreover, compared to that of the normal rats, the number of the glands was also decreased, and plasma cells and lymphocytes infiltration could be found in the lamina propria of stomach in model rats. The abnormal pathological changes of gastric glands and the histopathologic manifestation of model rats were obviously improved by folic acid treatment and WQD treatment. Furthermore, AB-PAS staining was used to detect the intestinal metaplasia. As shown in Figure 3, at 40 weeks, gastric intestinal metaplasia was appeared in the pylorus of model rats, which could be significantly attenuated by folic acid treatment and WQD treatment in a dose-dependent manner.

### 3.3. WQD Increased the Serum PGE2 Levels in the CAG Rats with Precancerous Lesion

**Figure 4:**
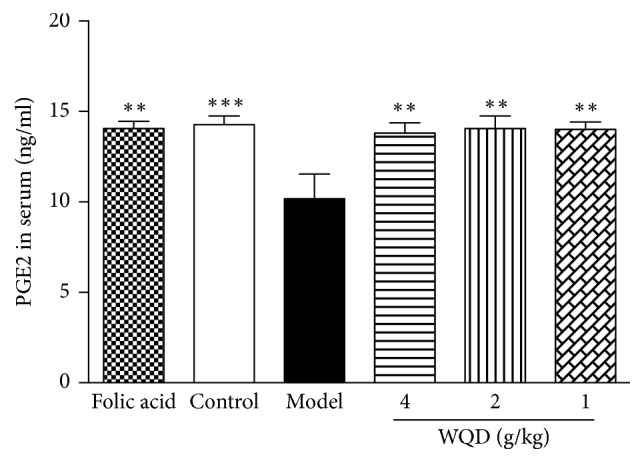
Effects of WQD on the serum PGE2 levels in model rats. Values were expressed as mean ± SD (n=6-8/group). Data were analyzed by one-way ANOVA assay. *∗∗*p < 0.01 vs model group.

As shown in Figure 4, compared with control rats, the serum PGE2 level was significantly decreased in model rats (p<0.001); WQD at 4, 2, and 1 mg/kg could all increase the serum PGE2 levels in model rats (p<0.01; p<0.01; p<0.01). Also, folic acid could also attenuate the decreased PGE2 in serum of model rats (p<0.01).

### 3.4. WQD Inactivated HIF-1 Signaling Pathway in CAG Rats with Precancerous Lesion

**Figure 5:**
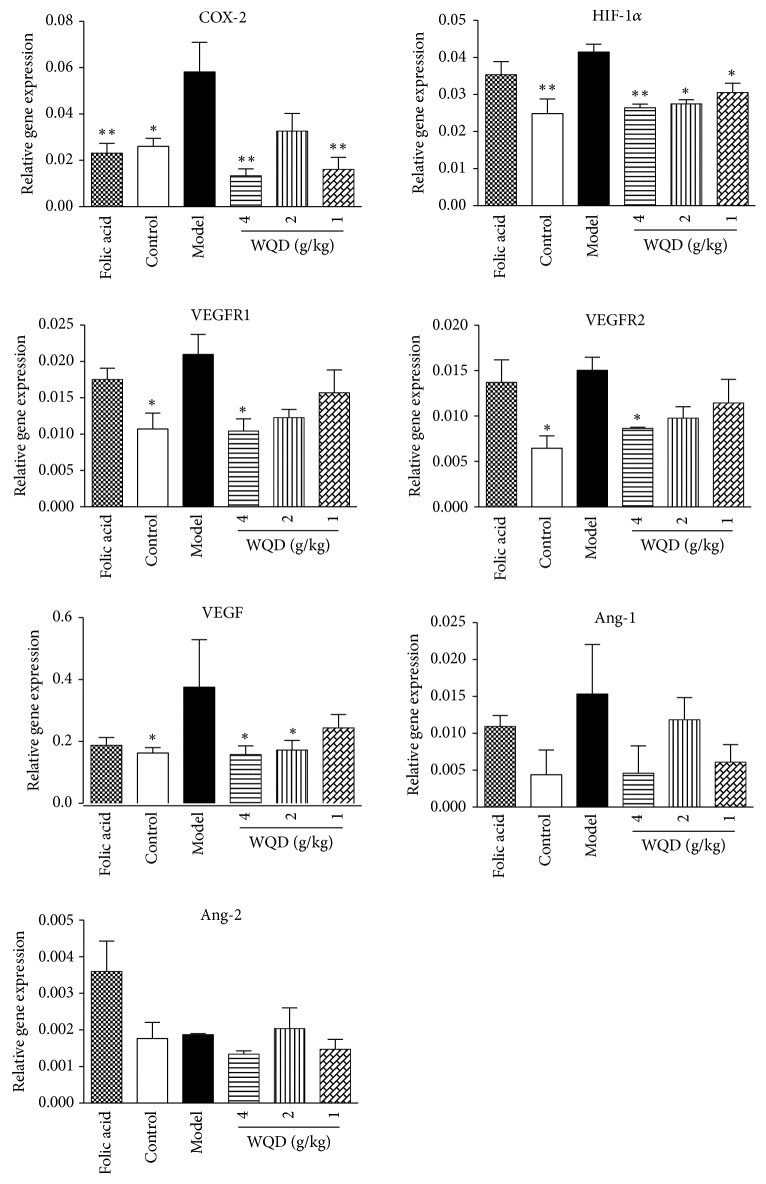
Effect of WQD on the gene expressions of COX-2, HIF-1*α*, VEGFR1, VEGFR2, Ang-1, and Ang-2 in the gastric tissue. Values were expressed as mean ± SD (n=6-8/group). Data were analyzed by one-way-ANOVA assay. *∗* p < 0.05. *∗∗* p < 0.01 vs model group.

At 40 weeks, the gene expression of COX-2, HIF-1*α*, VEGFR1, and VEGFR2 in the pylorus of model rats was all upregulated, compared with control rats. WQD could significantly downregulate the gene expression of HIF-1*α* in the pylorus of model rats (p<0.05). WQD at doses of 4 g/kg and 1g/kg could decrease the gene expression of COX-2 (p<0.01). Furthermore, the gene expression of VEGF, VEGFR1, and VEGFR2 in pylorus of model rats was apparently inhibited by WQD at 4 g/kg (p<0.05). However, the gene expression of Ang-1 and Ang-2 was not significant differences among control, model, WQD-treated, and folic acid treated rats. Folic acid could downregulate the gene expression of COX-2 (Figure 5).

**Figure 6:**
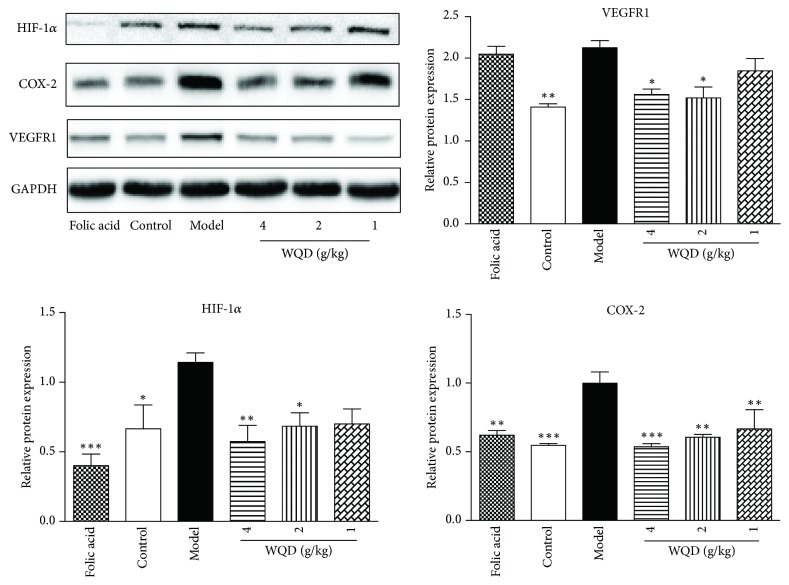
Effects of WQD on the relative protein expressions of COX-2, HIF-1*α*, VEGF and, VEGFR1 in the gastric tissue. Values were expressed as mean ± SD (n=6-8/group). Data were analyzed by one-way ANOVA assay. *∗* p < 0.05. *∗∗* p < 0.01, and *∗∗∗*p < 0.001 vs model group.

Compared with the control group, the protein expression of COX-2, HIF-1*α*, VEGF, and VEGFR1 in stomach tissue of model rats was significantly upregulated (Figure 6); however, WQD at doses of 4g/kg and 2g/kg could obviously downregulate the protein expression of HIF-1*α* (p<0.05). WQD at three doses and folic acid evidently decreased the protein expression level of COX-2 (p<0.01). WQD at doses of 4g/kg and 2g/kg but not folic acid could significantly downregulate the protein expression of VEGFR1 in the pylorus tissue of model rats.

### 3.5. WQD Affected Protein Expression of Ki67 and Cleaved Caspase 3 in Pylorus of CAG Rats with Precancerous Lesion

**Figure 7:**
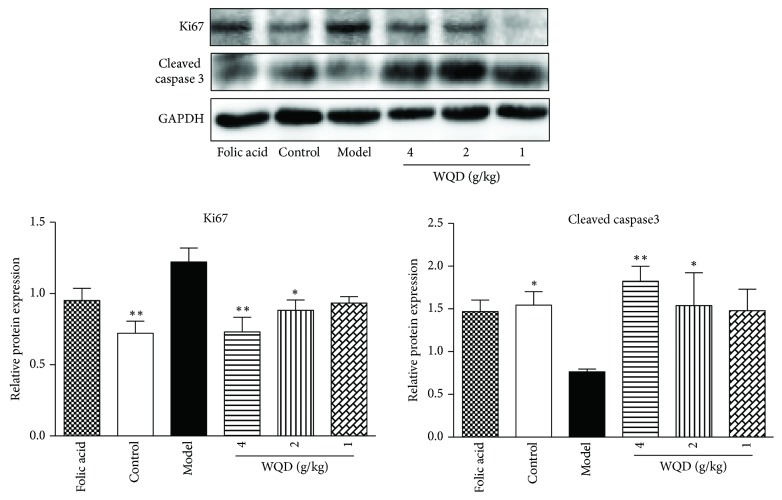
Effects of WQD on the protein expressions of Ki67 and Cleaved caspase 3 in the pylorus of CAG rats with precancerous lesion. Values were expressed as mean ± SD (n=6-8/group). Data were analyzed by one-way ANOVA assay. *∗* p < 0.05. *∗∗* p < 0.01 vs model group.

**Figure 8:**
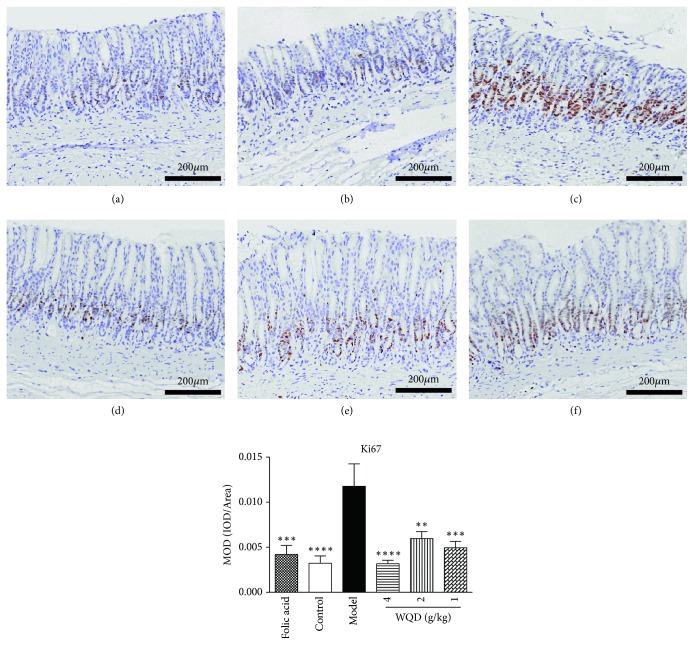
WQD treatment decreased protein expression of Ki67 detected by IHC staining in pylorus of CAG rats with precancerous lesion (100× magnifications). (a) Folic acid group, (b) Control group, (c) model group, (d) WQD 4 g/kg group, (e) WQD 2 g/kg group, and (f) WQD 1 g/kg group. Values were expressed as mean ± SD (n=6-8/group). Data were analyzed by one-way ANOVA assay. *∗∗*p < 0.01, *∗∗∗*p < 0.001, and *∗∗∗∗* p < 0.001 vs model group.

In this study, the balance of cell proliferation and apoptosis was also identified. WB results showed that, compared to control rats, the protein expression of Ki 67 in pylorus of model rats was significantly increased; however, protein expression of cleaved caspase 3 was significantly decreased. Both WQD and folic acid treatment could decrease the protein expression of Ki 67 but increase the protein expression of cleaved caspase 3 in pylorus of model rats (Figure 7). Moreover, IHC results also showed that protein expression of Ki 67 in the pylorus of model rats was obviously increased, compared with control rats, which could be decreased by WQD and folic acid treatment (Figure 8).

## 4. Discussion

Chronic atrophic gastritis is a common chronic digestive disease characterized by the loss of glands. At present, atrophic gastritis and intestinal metaplasia are thought to be one of the most important precancerous lesions. The incidence of chronic atrophic gastritis is about 10.9%. Gastric mucosal atrophy could significantly increase the risk of gastric cancer [3, 31]. In this study, parts of the gastric glands disappeared and the size of glands was different from each other; also intestinal metaplasia appeared in the gastric pylorus of CAG rats with precancerous lesion. WQD could significantly attenuate the gastric glands atrophy as well as intestinal metaplasia of gastric pylorus of CAG rats with precancerous lesion.

Traditional Chinese medicine treats CAG against Qi deficiency and blood stasis to obtain excellent therapeutic effect on CAG in clinic [13, 14]. Indeed, both CAG patients and rat models have angiogenesis and hematologic microcirculation disorders [32, 33]. In endoscopically, pale mucosa, nodular bulges, exposed vessels, and scattered bleeding points were exposed directly. Blood circulation disorder plays a critical role in the pathological process of CAG with precancerous lesions [34–36]. In the present study, the serious disturbed gastric microcirculation in stomach of CAG rats with precancerous lesion could be attenuated by WQD and folic acid treatment.

Epithelial cells play a key role in maintaining gastric mucosal homeostasis against a constant threat of inflammatory insult. Prostaglandin E2 (PGE2) is an important cell growth and regulatory factor and has the critical role in maintaining gastrointestinal epithelial barrier function. In the process of chronic inflammation, the decrease of PGE2 further interrupts the wound-healing process, thereby exacerbating the severity of the disease [37]. In present study, the serum level of PGE2 in CAG rats with precancerous lesion was greatly decreased, compared with control rats, indicating a serious gastric mucosal injury and inflammation, which was consistent with the increased expression of COX-2, gastric glands atrophy, and intestinal metaplasia in CAG rats. WQD could significantly increase the serum PGE2 secretion in CAG rats, indicating its potential inhibitory effect on gastric inflammation and the homeostasis of gastric mucosal epithelial barrier in CAG rats.

As HIF-1*α* signaling pathway plays a critical role in angiogenesis and hematologic microcirculation, the effect of WQD on gene and protein expression of HIF-1*α* signaling pathway was measured by qPCR and WB analysis. HIF-1*α* regulates angiogenesis-related genes against hypoxia [38, 39]. VEGF is one of the target genes of HIF-1*α* and is the primary cytokine to promote angiogenesis. HIF-1*α* expression is upregulated in response to hypoxic condition and enhances VEGF expression to specifically bind to specific receptors Flt-1 and KDR located on vascular endothelial cells, to promote endothelial cell growth, increase vascular permeability, and promote angiogenesis [10]. HIF-1*α* can also induce the expression of COX-2 in human endothelial cells to play the role in angiogenesis by multiple ways [40]. In this study, the increased protein levels of HIF-1*α*, COX-2, VEGF, and VEGFR1 in the CAG rats with precancerous lesion were significantly reduced by WQD treatment, suggesting WQD improve the gastric mucosal microcirculation and inhibit inflammation through regulating HIF-1*α* signaling pathway.

An imbalance between cell proliferation and cell death would result in tumor formation and growth [41]. And, hypoxia is a common characteristic in tumor microenvironment and could induce the upregulation of HIF-1*α*, which induces angiogenesis and also activates many other target genes mediating cell proliferation and apoptosis of cancer [42–44]. Cleaved caspase 3 is considered as an apoptosis marker and Ki 67 is considered as a proliferative cell marker in tumor formation [41, 45, 46]. Ki67 could be highly induced under hypoxia [46]. Indeed, HIF-1*α* could enhance cell proliferation and broke the balance between proliferation and apoptosis, and the increased expression of Ki67 and high level of HIF-1*α* in cancer predicts poor prognosis in many cancers, such as pancreatic cancer, colorectal cancer, breast cancer, and so on [44, 47–49]. In this study, the protein expression of Ki67 in the pyloric gastric mucosa of CAG was significantly increased, while the protein expression of cleaved caspase 3 was significantly decreased compared with that of control rats, indicating disturbed cell homeostasis of proliferation and apoptosis existed in pyloric gastric mucosa of CAG rats with precancerous lesions and also indicating the existence of atypical hyperplasia and intestinal metaplasia. And, WQD could enhance the protein expression of cleaved caspase 3 and decrease the protein expression of Ki67, indicating WQD could attenuate the disturbed cell homeostasis of proliferation and apoptosis in pyloric gastric mucosa in CAG rats with precancerous lesions.

## 5. Conclusions

WQD ameliorated CAG with precancerous lesions through improving the gastric mucosal blood microcirculation and attenuating imbalance of proliferation and apoptosis via regulating HIF-1*α* signaling pathway.

## Figures and Tables

**Table 1:** The sequences for forward and reverse primers used for qPCR.

Genes	Forward primer (5'-3')	Reverse primer (5'-3')
VEGF	CCTCTCCCTACCCCACTTCCT	CACTTTCTCTTTTCTCTGCCTCCAT
VEGFR1	TTGATGGTAGGCTGAGGGATG	AGATGTAACTGCCGAGGATGC
VEGFR2	GAGTTGGTGGAGCATTGGGAA	ATACAGGAAACAGGTGAGGTAGGCA
HIF-1*α*	CCCATTCCTCATCCATCAAACATT	CTTCTGGCTCATAACCCATCAACTC
COX-2	TGAAATATCAGGTCATCGGTGGAG	CATACATCATCAGACCCGGCAC
Ang-1	TTGGTTACTCGTCAGACATTCATC	TCTTCTTCTCTTTTTCCTCCCTTTA
Ang-2	AAGTCAACGCTGCCATCTTCC	GACCTTCCCCAACTCCACAGA
GAPDH	TCTCTGCTCCTCCCTGTTC	ACACCGACCTTCACCATCT

## Data Availability

The data used to support the findings of this study are available from the corresponding author upon request.

## Abbreviations

GC: Gastric cancer
CAG: Chronic atrophic gastritis
WQD: Weiqi decoction
MNNG: N-Methyl-N'-nitro-N-nitrosoguanidine
GMBF: Gastric mucosal blood flow
H&E: Hematoxylin and eosin
AB-PAS: Alcian blue and periodic acid-Schiff
IHC: Immunohistochemistry
HIF-1*α*: Hypoxia-inducible factor-1 alpha
COX-2: Cyclooxygenase-2
VEGF: Vascular endothelial growth factor
VEGFR1: VEGF receptor 1
VEGFR2: VEGF receptor 2
Ang-1: Angiopoietin 1
Ang-2: Angiopoietin 2
ELISA: Enzyme-linked immunosorbent assay
ECL: Electrochemiluminescence
ANOVA: One-way analysis of variance
DAB: Diaminobenzidine.
